# Spectral morphometric characterization of breast carcinoma cells

**DOI:** 10.1038/sj.bjc.6690257

**Published:** 1999-03

**Authors:** I Barshack, J Kopolovic, Z Malik, C Rothmann

**Affiliations:** Pathology Department, Sheba Medical Center, Tel-Hashomer, 52621, Israel; Microscopy Unit, Life Sciences Department, Bar-Ilan University, Ramat-Gan, 52900, Israel

**Keywords:** spectral morphometry, spectral similarity mapping, in situ and infiltrating ductal and lobular breast carcinoma

## Abstract

The spectral morphometric characteristics of standard haematoxylin and eosin breast carcinoma specimens were evaluated by light microscopy combined with a spectral imaging system. Light intensity at each wavelength in the range of 450–800 nm was recorded for 10^4^ pixels from each field and represented as transmitted light spectra. A library of six characteristic spectra served to scan the cells and reconstruct new images depicting the nuclear area occupied by each spectrum. Fifteen cases of infiltrating ductal carcinoma and six cases of lobular carcinoma were examined; nine of the infiltrating ductal carcinoma and three of the lobular carcinoma showed an in situ component. The spectral morphometric analysis revealed a correlation between specific patterns of spectra and different groups of breast carcinoma cells. The most consistent result was that lobular carcinoma cells of in situ and infiltrating components from all patients showed a similar spectral pattern, whereas ductal carcinoma cells displayed spectral variety. Comparison of the in situ and the infiltrating ductal solid, cribriform and comedo carcinoma cells from the same patient revealed a strong similarity of the spectral elements and their relative distribution in the nucleus. The spectrum designated as number 5 in the library incorporated more than 40% of the nuclear area in 74.08% of the infiltrating lobular cells and in 13.64% of the infiltrating ductal carcinoma cells (*P* < 0.001). Spectrum number 2 appeared in all infiltrating ductal cells examined and in none of the lobular cells. These results indicate that spectrum number 5 is related to infiltrating lobular carcinoma, whereas spectrum number 2 is characteristic for infiltrating ductal carcinoma cells. Spectral similarity mapping of central necrotic regions of comedo type in situ carcinoma revealed nuclear fragmentation into defined segments composed of highly condensed chromatin. We conclude that the spectral morphometric features found for lobular and ductal cell populations may serve future automated histological diagnostics. © 1999 Cancer Research Campaign


*British Journal of Cancer* (1999) **79**(9/10), 1613–1619
© 1999 Cancer Research Campaign
Article no. bjoc.1998.0257

### Spectral morphometric characterization of breast carcinoma cells

**I Barshack[1], J Kopolovic[1], Z Malik[2] and C Rothmann[2]**

[1]Pathology Department, Sheba Medical Center, Tel-Hashomer 52621, Israel; [2]Microscopy Unit, Life Sciences Department, Bar-Ilan University, Ramat-Gan 52900, Israel

**Summary** The spectral morphometric characteristics of standard haematoxylin and eosin breast carcinoma specimens were evaluated by light microscopy combined with a spectral imaging system. Light intensity at each wavelength in the range of 450–800 nm was recorded for $10^4$ pixels from each field and represented as transmitted light spectra. A library of six characteristic spectra served to scan the cells and reconstruct new images depicting the nuclear area occupied by each spectrum. Fifteen cases of infiltrating ductal carcinoma and six cases of lobular carcinoma were examined; nine of the infiltrating ductal carcinoma and three of the lobular carcinoma showed an in situ component. The spectral morphometric analysis revealed a correlation between specific patterns of spectra and different groups of breast carcinoma cells. The most consistent result was that lobular carcinoma cells of in situ and infiltrating components from all patients showed a similar spectral pattern, whereas ductal carcinoma cells displayed spectral variety. Comparison of the in situ and the infiltrating ductal solid, cribriform and comedo carcinoma cells from the same patient revealed a strong similarity of the spectral elements and their relative distribution in the nucleus. The spectrum designated as number 5 in the library incorporated more than 40% of the nuclear area in 74.08% of the infiltrating lobular cells and in 13.64% of the infiltrating ductal carcinoma cells ($P < 0.001$). Spectrum number 2 appeared in all infiltrating ductal cells examined and in none of the lobular cells. These results indicate that spectrum number 5 is related to infiltrating lobular carcinoma, whereas spectrum number 2 is characteristic for infiltrating ductal carcinoma cells. Spectral similarity mapping of central necrotic regions of comedo type in situ carcinoma revealed nuclear fragmentation into defined segments composed of highly condensed chromatin. We conclude that the spectral morphometric features found for lobular and ductal cell populations may serve future automated histological diagnostics.

**Keywords:** spectral morphometry; spectral similarity mapping; in situ and infiltrating ductal and lobular breast carcinoma

The histological identification of ductal and lobular carcinoma has proven to be essential for evaluation of patient prognosis and determination of treatment (Toikkanen and Jensuu, 1990; Ellis et al, 1992; Eskelinen et al, 1992). Ductal and lobular lesions may present similar histopathological appearances, as observed in their mode of invasiveness (Azzopardi et al, 1982). At the same time, morphometric and stereological methods have shown that changes in nuclear structure and chromatin patterns are a significant aid to histological confirmation (Stenkvist et al, 1978; Cornelisse et al, 1984; Dawson et al, 1991; Ladekarl and Sorensen, 1993). Cells of infiltrating lobular carcinoma are usually smaller than those of ductal carcinoma, less pleomorphic and have fewer mitotic figures, whereas infiltrating ductal carcinoma cells have more prominent nucleoli (Fisher et al, 1975). The tumours also have some differences in clinical behaviour and in the pattern of metastasis. Lobular carcinoma is more multifocal and bilateral than ductal carcinoma (Azzopardi et al, 1982), and patient survival expectancy is usually better (DiConstanzo et al, 1990; duToit et al, 1991). However, ductal lesions constitute close to 80% of breast carcinoma cases (Toikkanen and Jensuu, 1990). Some histological types of in situ ductal carcinoma have been recognized and subdivided into comedo, cribriform, solid and micropapillary types on the basis of architectural pattern, cellular plemomorphism and nuclear hyperchromasia (Page and Anderson, 1987; Lagios, 1990).

Characterization of nuclear features by different techniques is used for the determination of diagnosis and prognosis. Quantitative estimation of various histopathological parameters such as two-dimensional estimates of nuclear profile area, nuclear profile densities and mitotic profile numbers have been shown to correlate with differentiation and prognosis. Alterations in nuclear structure are the morphological hallmark of cancer diagnosis. Nuclear size, shape, chromatin pattern and nucleolar size and number have all been reported to change in breast cancer (Pienta and Coffey, 1991). However, heterogeneity in morphology and biology of tumours belonging to the same classification group has been found to be a prominent feature of breast cancer (Heppner, 1984; Stoll, 1986).

Ladekarl and Sorensen (1993) found that the mean three-dimensional nuclear size, the mean nuclear profile area and the mitotic index were all significantly larger in ductal than in lobular carcinomas, whereas the mean nuclear density index was smaller in ductal carcinoma. Yu et al (1995) also identified some distinct nuclear features useful in the differentiation of infiltrating ductal and lobular carcinoma.

Spectral morphometry is a newly developed method used here for the evaluation of histopathological features. This method provides multipixel spectral information which otherwise is impossible to obtain. The spectral measurements are based on Fourier transform spectroscopy, a technique of proven capability for spectral analysis (Garini et al, 1996*a*). This technique has

*Received 3 March 1998*
*Revised 20 May 1998*
*Accepted 28 May 1998*

*Correspondence to:* Z Malik

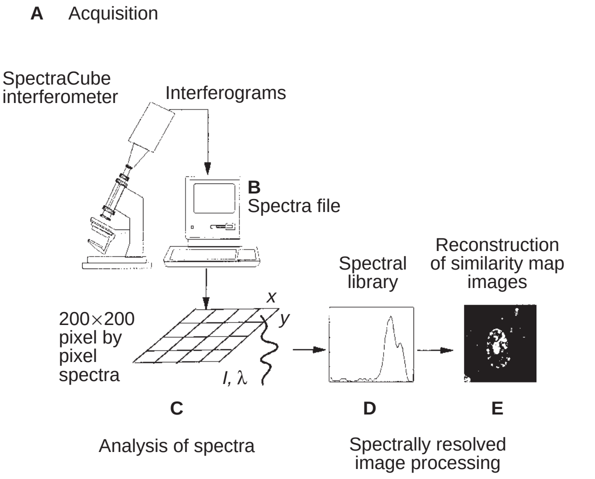

**Figure 1** Simplified diagram of the SpectraCube method. (**A**) The light emitted from the sample is collected and proceeds to the Sagnac interferometer, the image is focused on the CCD array, yielding the interferogram at each pixel. (**B**) Mathematical transformation of the data into a spectral image cube by fast Fourier transform. (**C**) The 'cube' appellate signifies the two spatial dimensions of a flat sample (*x* and *y*), and the third wavelength dimension. (**D**) A single spectrum may serve for spectral similarity mapping of the whole image producing a grey scale image based on the degree of similarity (**E**)

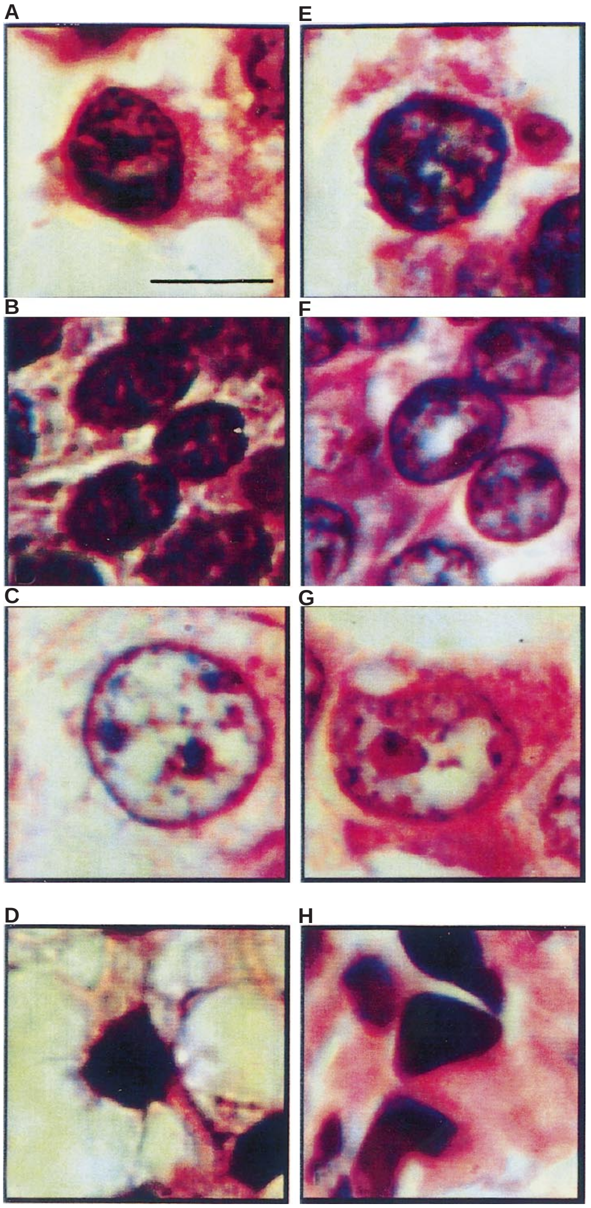

**Figure 2** Spectral imaging of H and E-stained breast carcinoma cells. (**A**–**C**) H and E-stained cells of in situ carcinoma (solid, cribriform and comedo respectively). (**E**–**G**) Infiltrating ductal carcinoma from the same cases as in **A**–**C** respectively. In situ lobular carcinoma (**D**) and infiltrating lobular carcinoma (**H**). Bar = 8 μm already been used in a large number of biological fields. The subcellular localization of porphyrins in treated cancer cells was determined using the known fluorescence spectra of the porphyrins as a marker for tracing (Malik et al, 1996*a*, 1997). Nuclear organization in normal erythropoiesis and apoptosis was studied by the transmitted light spectra of stained cytological specimens (Rothmann et al, 1997). In addition, a novel karyotyping method was developed using the SKY technique (Garini et al, 1996*b*) to detect chromosomal abnormalities in solid tumours (Schrock et al, 1996) and leukaemias (Veldman et al, 1997). This study is the first attempt to identify and evaluate human tumour cells using this technique.

The purpose of the present study was to determine spectral morphometric distinctions between ductal and lobular carcinoma cells stained with haematoxylin and eosin (H and E) by multipixel spectroscopy. A comparison between infiltrating and in situ components of these tumours is also described.

### MATERIALS AND METHODS

Twenty-one cases, 15 of which were infiltrating ductal carcinoma (histological grade II) and six of which were infiltrating lobular carcinoma, were randomly selected from the surgical pathology material of the Sheba Medical Center, Department of Pathology. The material consisted of lumpectomy and mastectomy specimens. The use of grade II tumours eliminated the wide variability that exists within each group of infiltrating ductal carcinoma.

### Histology

Formalin-fixed paraffin-embedded tissues were cut to 4-μm-thick sections, and all sections were stained by H and E at the same time under standard conditions. Blocks from previously frozen tissues were excluded. All sections were reviewed by two independent pathologists and a diagnosis was made by the criteria described by the WHO (Scarff and Torloni, 1968; Hartman et al, 1981). The histological grading was made using the system of Bloom and Richardson, which considers tubular formation, nuclear pleomorphism and frequency of mitoses (Gallager and Hutter, 1978). All cases were searched for foci of in situ carcinoma using the system of Lennington et al (1994). Nine out of 15 cases of infiltrating ductal carcinoma and three out of six cases of infiltrating lobular carcinoma were associated with an in situ component.

### Fourier-transform multipixel spectroscopy system for microscopy

Systematic random sampling (Gundersen and Jensen, 1987) was performed on the H and E-stained breast carcinoma cells. The first

 © Cancer Research Campaign 1999

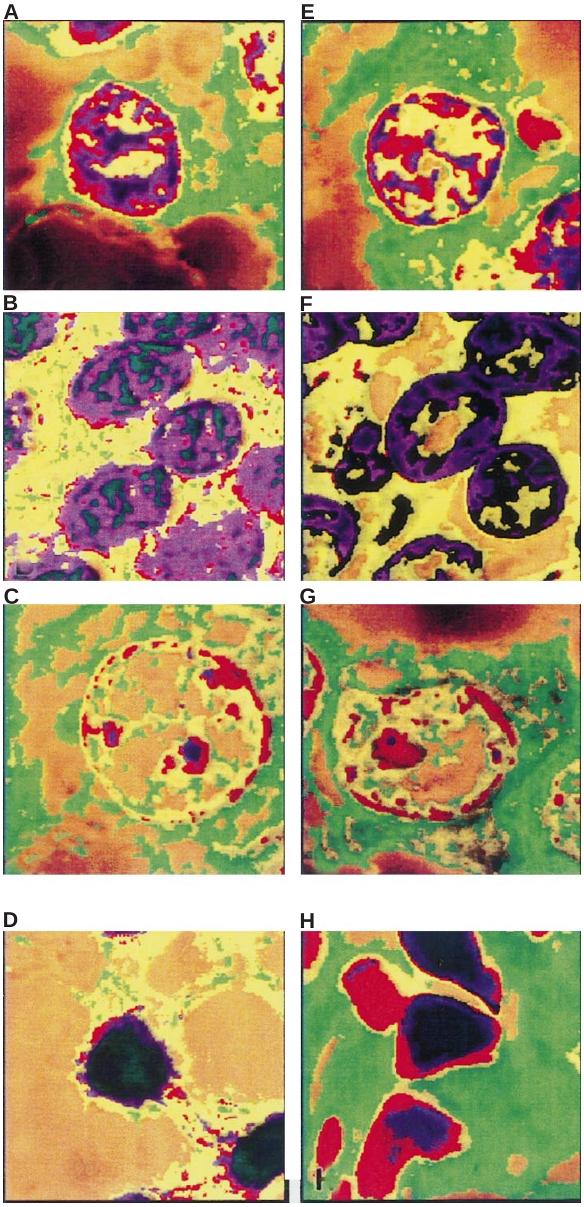

**Figure 3** Spectral classification of H and E-stained breast carcinoma cells. (**A**–**C**) Spectrally classified images of in situ carcinoma cells, solid, cribriform and comedo respectively, represented here by a single case for each type. Each coloured domain represents a spectrally similar region. (**E**–**G**) Spectrally classified images of infiltrating duct carcinoma from the same cases as in **A**–**C** respectively. (**D** and **H**) Spectrally classified images of in situ lobular carcinoma and infiltrating lobular carcinoma respectively

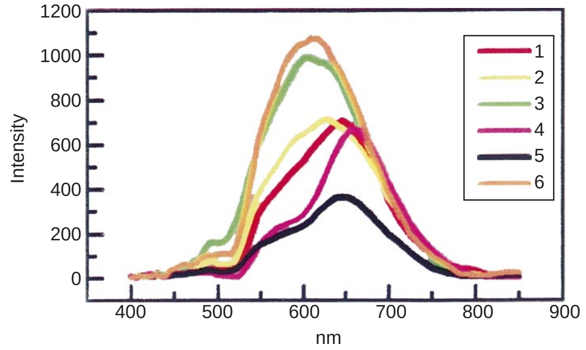

**Figure 4** Spectral library. Six separate spectral groups representing different regions of the cell were assigned an arbitrary colour: (1) red for nucleolus; (2) yellow for interchromosomal region; (3) green for cytoplasm; (4) and (5) are black and magenta, respectively, representing two distinct chromatin domains, the former less condensed than the latter; (6) orange for a low-density space. Each spectral group is an average of 24 spectra sampled from different cells field of vision was selected randomly and subsequent fields were adjacent to the first one. Each field was imaged and processed. Light intensity at each wavelength in the range of 450–800 nm was recorded for $10^4$ pixels from each field and represented as transmitted light spectra.

Spectral imaging was performed using the SpectraCube SD-200 (Applied Spectral Imaging, Migdal HaEmek, Israel). The SpectraCube system consists of an interferometer situated in the parallel beam between an objective lens (infinity corrected) and a lens equivalent to an eyepiece, whose purpose is to form an image on a CCD camera. The light beam passing through the specimen is split in the interferometer in opposite directions, and is united again at the exit with an optical path difference (OPD) which is a function of the angle between the incoming beam and the interferometer itself. The OPD arises because for non-zero angles the two beams undergo different optical paths in the beam-splitter. The inherent mechanical stability of this interferometer allows the Fourier technique to be successfully applied to the visible spectral region. The measurement is carried out by recording successive CCD frames in synchronization with the steps of the motor used to rotate the collimated beam, so that the instantaneous OPD is known for every pixel in every recorded frame and can be used in the fast Fourier transform (FFT) calculation (Malik et al, 1996*a*, 1996*b*). During a measurement (20 s), each pixel of the CCD (512 × 512) is collecting the interferogram, which is then Fourier transformed to give the spectrum (Figure 1).

In spectral imaging, each pixel is actually one of several tens of thousands of microspectrometers, acting simultaneously and independently. As a result, spectral imaging acquires a so-called cube whose appellate signifies the two spatial dimensions of a flat sample (*x* and *y*), and the third wavelength dimension. The calculated pixel size in a spectral image is 0.04 $\mu m^2$. The spectral resolution (FWHM, full width at half maximum) is 5 nm at 400 nm (12 nm at 600 nm) and the spectral range (more than 5% response) is 400–1000 nm (Garini et al, 1996*a*).

### Spectral similarity mapping

Spectral analysis of a 'test set' of breast carcinoma cells, three cells from each type of tumour, revealed six different spectral regions composing the cells: nucleolus, two distinct chromatin regions of the nucleus, an interchromosomal region, cytoplasm and a low-density space. To segregate these regions, we used the spectral similarity mapping function, which calculates the differences between area-integrals of one reference spectrum with respect to all the other spectra constituting an image. The comparison algorithm used in this work for similarity mapping is defined by the following function:

$$f_{x,y} = \left(\int_{\lambda 1}^{\lambda 2} [I_{x,y}(\lambda) - I_o(\lambda)]^2 \, d\lambda\right)^{1/2}$$

© Cancer Research Campaign 1999

$I_{x,y}$ is the spectrum of the pixel of coordinates *x* and *y* of the image, as a function of $\lambda$. The integral stands for an interval over a predetermined spectral range $\lambda_1 - \lambda_2$ and $I_0$ is the reference spectrum. The reconstructed similarity map image was composed of bright and grey pixels and reveals the degree of similarity between the spectra. The brighter the pixel, the more the two spectra are alike.

The average spectra of each region, sampled from 24 different cells, were used to construct a spectral library, and we used each spectrum in the spectral library as a reference spectrum. Spectral similarity mapping of a cell produced similarity maps for each of the spectra in the library. All spectrally similar regions were assigned an arbitrary colour: reference spectrum 1 sampled from the nucleolus was assigned red; reference spectrum 2 from the interchromosomal region, yellow; reference spectrum 3 from the cytoplasm, green; reference spectra 4 and 5 representing two distinct chromatin domains, the former less condensed than the latter, are black and magenta respectively; reference spectrum 6 of the low-density space was assigned the colour orange. A colour array was defined for each type of tumour based on the classified images of the 'test set' of tumours. The spectral library served to scan 120 cells from 15 cases of in situ ductal carcinoma, 72 cells from nine cases of infiltrating ductal carcinoma, 48 cells from six cases of in situ lobular carcinoma and 24 cells from three cases of infiltrating lobular carcinoma (eight cells from each case). The classified image of each cell was compared with the colour arrays in order to determine its type.

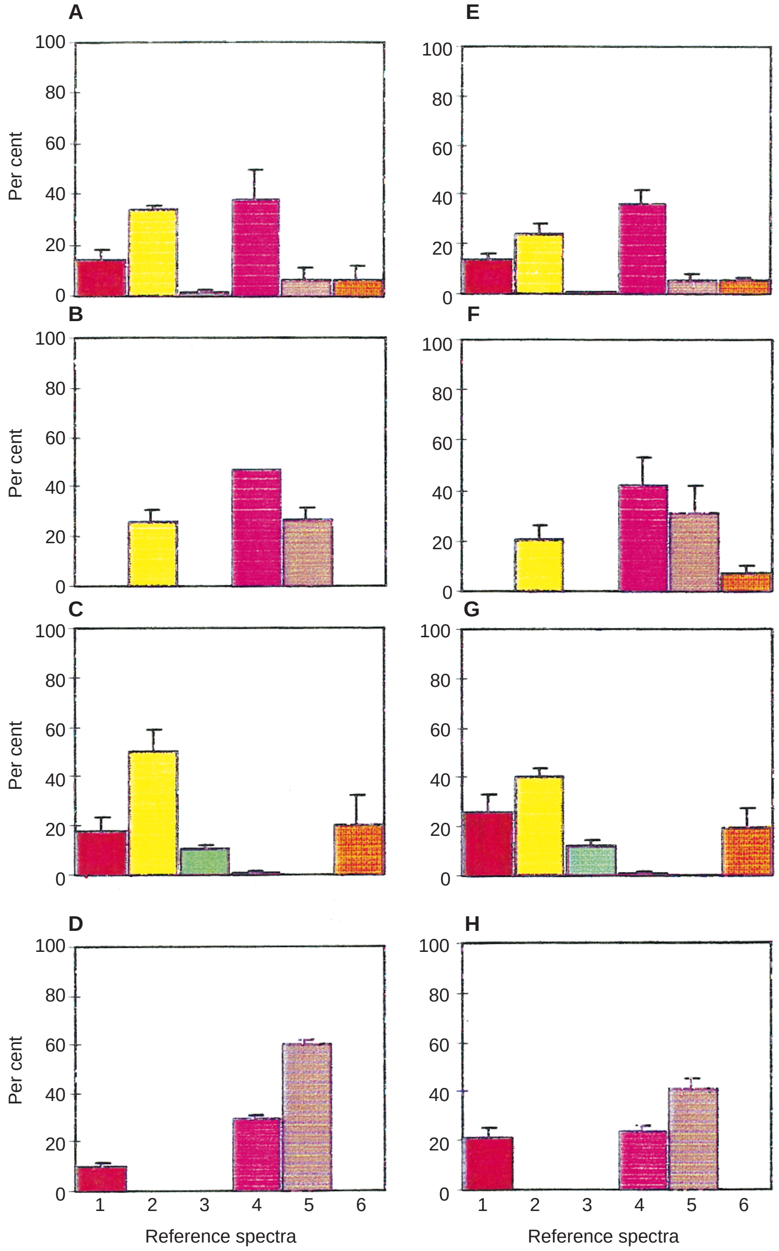

**Figure 5** Spectral arrays. The area of each spectrally similar region in the nucleus (shown in Figure 3 A–H) was calculated and represented as the percentage of the nuclear area ±s.e. (**A**–**C**) Spectral arrays of the three histological types of in situ carcinoma, solid, cribriform and comedo, represented by a single case for each type. (**E**–**G**). Infiltrating ductal carcinoma from the same patients respectively. (**D** and **H**) In situ and infiltrating lobular carcinoma cells respectively

 *© Cancer Research Campaign 1999*

The spectral similarity mapping was performed on normalized spectra to eliminate intensity variations. The variation in dye concentration was overcome by standardization of all the spectra files according to a standard spectrum of extracellular matrix. The spectra of an examined specimen were multiplied by the ratio between the spectrum of extracellular matrix in a standard stained specimen and the spectrum of extracellular matrix in the examined specimen.

### RESULTS

Spectral imaging was used to measure the light intensity at any wavelength over $10^4$ pixels of the different cellular compartments of breast carcinomas. Figure 2 A–H shows eight cells stained by H and E, representing areas of infiltrating and of in situ ductal carcinoma (solid, cribriform and comedo), and of in situ and infiltrating lobular carcinoma. Chromatin domains in these nuclei are demarcated by stain–chromatin complexes seen as multicoloured sites. The cells of Figure 2 stained by H and E show differences in nuclear size, structure and chromatin condensation.

The classified spectral map for each image is shown in Figure 3A–H, revealing the distinct spectral regions in each cellular compartment; each coloured region corresponds to one spectrum of the library of Figure 4. Thus, the reconstructed images (Figure 4A–H) represent spectrally similar regions with respect to the reference spectra of the library. The area of all spectrally similar regions of the nucleus was calculated (Figure 5A–H). The nuclear area of the in situ solid, cribriform and comedo cells displayed differing distributions of spectrally similar regions; cells from different patients displayed distinct spectral characteristics. The comparison of in situ and infiltrating cells from the same patient revealed a strong resemblance of the spectral elements and their distribution in the nucleus (Figure 5A–C compared with Figure 5E–G). The classification maps of in situ and infiltrating lobular carcinoma reveal an almost identical distribution of spectrally similar regions for all cells examined (Figure 5D and H).

These findings lead to the determination of some spectral morphometric parameters. Evaluation of the figures representing lobular carcinoma reveals that the presence of the spectral arrays 1, 4 and 5 is related to the lobular carcinoma cells, whereas spectrum 2 is related to all ductal cells. Statistical analysis performed on the spectral arrays 1, 4 and 5 in infiltrating duct and lobular carcinoma revealed that the spectrum designated in our library as number 5 incorporated more than 40% of the nuclear area in 74.08% of the infiltrating lobular cells and in 13.64% of the infiltrating ductal carcinoma cells ($P < 0.001$). Thus, the difference in the appearance of spectrum number 5 is statistically significant between the two infiltrating histological tumours. However, the difference in appearance of spectrum number 1 (above 24% of nuclear area) and spectrum number 4 (above 34% of nuclear area) between infiltrating ductal and lobular carcinoma cells was not statistically significant. In addition, spectrum number 2 appeared in all infiltrating ductal cells examined and in none of the lobular cells.

Cells sampled from the central necrotic region of comedo type in situ carcinoma (Figure 6A) were analysed by the SpectraCube. Spectral classification distinguished between the distinct regions comprising the highly condensed nuclei, thus revealing nuclear breakage into defined fragments (Figure 6B).

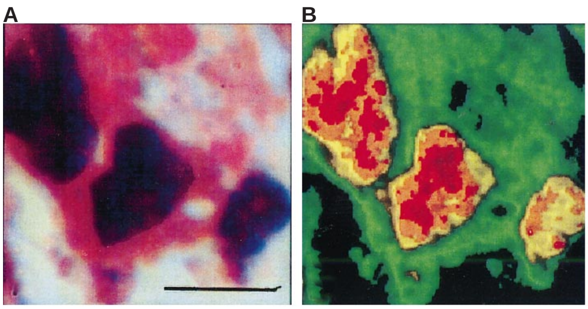

**Figure 6** Spectral imaging of necrotic cells. (**A**) Cells sampled from the central necrotic region of in situ carcinoma, comedo type, were analysed by the SpectraCube. (**B**) Spectral classification of the distinct regions comprising the highly condensed nuclei displaying chromatin fragments. Bar = 8 μm

### DISCUSSION

A novel spectral imaging system was used for characterization of standard H and E-stained breast carcinoma specimens. Specific patterns of spectra could be correlated with different groups of breast carcinoma cells. Nuclear structure and chromatin condensation in infiltrating ductal and lobular breast carcinoma and in the in situ component of each were determined.

Previous studies have proven that, besides mitosis counting, characterization of nuclear features, chromatin structure and nucleolus organization are the most important morphometric parameters for the determination of diagnosis and prognosis of breast cancer (Cornelisse et al, 1984; Gilchrist et al, 1985; Dawson et al, 1991). Quantitative estimation of various parameters such as three-dimensional estimates of nuclear mean volume (Ladekarl, 1995), two-dimensional estimates of nuclear mean area, average area of chromatin region per nucleus and the number of central chromatin regions per nucleus have been shown to correlate with differentiation and prognosis in breast cancer (Komitowski and Janson, 1990). The 3-D reconstruction of nuclear images allows visualization of chromatin-clustering regions, which were found to correlate with tumour aggressiveness (Komitowski et al, 1993). The nucleolar organizing region (NOR) index has recently been re-evaluated and redefined as a prognostic factor for breast cancer patient survival (Smith and Crocker, 1988). The use of a single set of variables for morphometric diagnosis introduces classification errors due to overlap (Baak and Oort, 1983), which can be overcome by adding other morphometric parameters such as spectral information of stain–macromolecule complex distribution in the cell.

The SpectraCube, the recently developed analytical spectral imaging system can measure transmittance and absorbance spectra of images studied by light microscopy. By comparing the spectrum of each pixel in the specimen with a selected reference spectrum, the spatial distribution of stained macromolecules possessing the characteristics of the reference spectrum can be determined (Malik et al, 1996*b*). Galbraith et al (1980) have shown that the differential colouration of cell structures can be explained in terms of varying proportions of dye complex with the macromolecules. By adopting computerized spectroscopic microanalysis of nuclear chromatin granularity, Spina et al (1992) showed a significant distinction between benign and malignant breast cells.

Our technique has advantages over the systems already used for image analysis. In addition to standard morphometric parameters, spectra (400–850 nm) are obtained for each pixel of an image, providing more information than conventional grey scale image

© Cancer Research Campaign 1999

analysis (Stenkvist et al, 1978) even when the latter is used in combination with colour filters (Wells et al, 1992). The transformation from a fully coloured image to a grey scale image, in morphometric methods, introduces some inaccuracy, which is totally eliminated by the SpectraCube. A coloured image displaying closely related wavelengths with the same intensity might appear similar in a grey scale image. However, spectral imaging differentiates between closely related colours even when the total intensity is similar and is thus more sensitive and specific.

In the present study, nuclear area classification based on the spectral similarity of ductal and lobular carcinoma cells of the breast was accomplished with the use of a spectral reference library. Lobular carcinoma cells from all cases displayed similar spectral arrays, whereas variable spectral arrays were obtained from the ductal carcinoma cells of all patients. The results indicated that spectrum number 5 is related to infiltrating lobular carcinoma, whereas spectrum number 2 is characteristic for infiltrating ductal carcinoma cells. However, there was a clear similarity between the spectra of infiltrating cells and the in situ component of the same patient also known to be similar in mean size (Ladekarl and Sorensen, 1993). These results correlate well with previous morphological data (Azzopardi et al, 1982; DiConstanzo et al, 1990).

We propose a new model for sorting different cell types by identification of spectral morphometric parameters using a 'spectral library'. Distinct patterns of spectra characterize different groups of cells. We believe that spectral morphometric analysis should serve as an additional tool for conventional morphometric methods with respect to the differential diagnosis of ductal and lobular carcinomas. Spectral analysis is considered to be an objective tool for characterization and comparison between different cells in histopathology (Farmer et al, 1996). The combination of several quantitative histopathological variables obtained by morphometric and spectral analysis would probably improve the discriminative ability of the pathologist. We have to bear in mind that much more work has to be carried out to establish the results. Systematic random sampling of the whole tumour area can be more efficient than the present technique. Spectral characteristics may be of primary importance when the biopsy contains only a small number of tumour cells, as in a core needle biopsy. Moreover, spectral imaging can serve for the analysis of different states of cell death processes such as apoptosis or necrosis, as shown for the in situ comedo type component.

### ACKNOWLEDGEMENTS

The authors gratefully thank Ms Judith Hanania for her help in editing the manuscript and Mr Jacob Langsam for his skilful assistance. This study was supported by a grant of Applied Spectral Imaging, Migdal HaEmek, Israel and by a grant from the Israeli Ministry of Health (to ZM and JK).

### REFERENCES

Azzopardi JG, Chepick OF, Hartmann WH, Jafarey NA, Lombart-Bosch A and Ozello L (1982) The World Health Organization histological typing of breast tumors 2nd ed. *Am J Clin Pathol* **78**: 806–816

Baak JPA and Oort J (1983) A manual of morphometry. In *Diagnostic Pathology*. Baak JPA and Oort J (eds). Springer-Verlag: Berlin

Cornelisse CJ, de Konig HR, Moolenaar AJ van de Velde CJ and Ploem JS (1984) Image and flow cytometric analysis of DNA content in breast cancer; relation to estrogen receptor content and lymph node involvement. *Anal Quant Cytol Histol* **4**: 9–18

Dawson AE, Austin RE and Weinberg DS (1991) Nuclear grading of breast carcinoma by image analysis. Classification by multivariate and neural network analysis. *Am J Clin Pathol* **95**: S29–S37

DiConstanzo D, Rosen PP, Gareen I, Franklin S and Lesser M (1990) Prognosis in infiltrating lobular carcinoma: an analysis of "classical" and variant tumors. *Am J Surg Pathol* **14**: 12–23

Ellis IO, Galea M, Broughton N, Locker A, Blaney RW and Elston C (1992) Pathological prognostic factors in breast cancer: II Histological type; relationship with survival in a large study with long term follow-up. *Histopathology* **20**: 479–489

Eskelinen M, Lipponen P, Papinaho S, Aaltomaa S, Kosma Vm and Klemi P (1996) DNA flow cytometry, nuclear morphometry, mitotic indices and steroid receptors as independent prognostic factors in female breast cancer. *Int J Cancer* **51**: 555–561

Farmer ER, Gonin R and Hanna MP (1996) Discordance in the histopathologic diagnosis of melanoma and melanocytic nevi between expert pathologists. *Hum Pathol* **27**: 528–531

Fisher ER, Gregorio RM and Fisher B (1975) The pathology of invasive breast cancer. A syllabus derived from findings of the national surgical adjuvant breast project (protocol no. 4). *Cancer* **36**: 1–85

Galbraith W, Marshall PN and Bacus JW (1980) Microspectrophotometric studies of Romanowsky stained blood cells. I. Subtraction analysis of a standardized procedure. *J Microsc* **119**: 313–330

Gallager HS and Hutter RVP (1978) Pathology and pathogenesis of breast cancer. In *The Breast*. Gallager HS, Leis HP, Snydcran RK, Urban IA (eds), p. 49. CV Mosby: St Louis

Garini Y, Katzir N, Cabib D, Buckwald RA, Soenksen D and Malik Z (1996*a*) Spectral Bio-Imaging. In *Fluorescence Imaging Spectroscopy and Microscopy*. Wang XF, Herman B (eds), p. 87. Wiley and Sons: New York

Garini Y, MacvM, du Manoir S, Buckwald RA, Lavi M, Katzir N, Wine D, Bar-Am I, Schrock E, Cabib D and Ried T (1996*b*) Spectral karyotyping. *Bioimaging* **4**: 64–72

Gilchrist KW, Kalish L, Gould VE, Hirschl S, Imbriglia JE, Levy WM, Patchefsky AS, Penner DW, Pickren J, Roth JA, Schinella RA, Schwart IS and Wheeler JE (1985) Inter-observer reproducibility of histopathological features in stage II breast cancer: an ECOG study. *Breast Cancer Res Treat* **5**: 3–10

Gundersen HSG and Jensen EB (1987) The efficiency of systematic sampling in stereology and prediction. *J Microsc* **147**: 229–234

Hartmann WH, Ozzello L, Sobin LH and Stalsberg H (1981) *Histologic Typing of Breast Tumors*. World Health Organization: Geneva

Heppner GH (1984) Tumor heterogeneity. *Cancer Res* **44**: 2259–2265

Komitowski DD and Janson CP (1990) Quantitive features of chromatin structure in the prognosis of breast cancer. *Cancer* **65**: 2725–2730

Komitowski DD, Hart MM and Janson CP (1993) Chromatin organization and breast cancer prognosis; two dimensional and three dimensional image analysis. *Cancer* **72**: 1239–1246

Ladekarl M and Sorensen FB (1993) Quantitive histopathological variables in in situ and invasive ductal carcinoma of the breast. *APMIS* **101**: 895–903

Ladekarl M (1995) Quantitative histopathology in ductal carcinoma of the breast. Prognostic value of mean nuclear size and mitotic counts. *Cancer* **75**: 2114–2122

Lagios MD (1990) Duct carcinoma in situ pathology and treatment. *Surg Clin North Am* **70**: 853–871

Lennington WJ, Jensen RA, Dalton LW and Page DL (1994) Ductal carcinoma in situ of the breast. Heterogeneity of individual lesions. *Cancer* **73**: 118–124

Malik Z, Dishi M and Garini Y (1996*a*) Fourier transform multipixel spectroscopy and spectral imaging of protoporphyrin in single melanoma cells. *Photochem Photobiol* **63**: 608–614

Malik Z, Cabib D, Buckwald RA, Talmi Y, Garini Y and Lipson SG (1996*b*) Fourier transform multipixel spectroscopy for quantitative cytology. *J Microsc* **182**: 133–140

Malik Z, Amit I and Rothmann C (1997) Subcellular localization of sulfonated tetraphenyl porphines in colon carcinoma cells by spectrally resolved imaging. *J Photochem Photobiol* **65**: 389–396.

Page DL and Anderson TG (1987) *Diagnostic Histopathology of the Breast*. Churchill Livingstone: Edinburgh

Pienta KJ and Coffey DS (1991) Correlation of nuclear morphometry with progression of breast cancer. *Cancer* **68**: 2012–2015

Rothmann C, Cohen AM and Malik Z (1997) Chromatin condensation in erythropoiesis resolved by multi-pixel spectral imaging: differentiation versus apoptosis. *J Histochem Cytochem* **45**: 1097–1108

© Cancer Research Campaign 1999

Scarff RW and Torloni H (1968) Histologic typing of breast tumors. In *International Classification of Tumors*, Vol. 2, Scarff RW and Torloni H (eds), p. 19. World Health Organization: Geneva

Schrock E, du Manoir S, Veldman T, Schoell B, Wienberg J, Ferguson-Smith MA, Ning Y, Ledbetter DH, Bar-Am I, Soenksen D, Garini Y and Ried T (1996). Multicolor spectral karyotyping of human chromosomes. *Science* **273**: 494–497

Smith R and Crocker J (1988) Evaluation of nucleolar organizer region associated proteins in breast malignancy. *Histopathology* **12**: 113–125

Spina D, Disanto A, Luzi P, Tosi P, Gallorini M, Mouthon AM, Kraft R and Cottier H (1992) Novel, contrast gradient-oriented, automated chromatin texture analysis. Feasibility study on nuclei from benign and malignant breast epithelial cell lines in fine needle aspirates. *Virchows Arch B Cell Pathol* **62**: 119–124

Stenkvist B, Westman-Naeser S, Vegelius J, Holmquist J, Nordin B, Bengtsson E and Eriksson O (1978) Computerized nuclear morphology as an objective method for characterizing human cancer cell populations. *Cancer Res* **38**: 4688–4977

Stoll BA (1986) Components of a prognostic index. In *Breast Cancer Treatment and Prognosis*, Stoll BA (ed.), p.115. Blackwell Scientific Publications: London

Toikkanen S and Jensuu H (1990) Prognostic factors and long-term survival in breast cancer in a defined urban population. *APMIS* **98**: 1005–1014

du Toit RS, Locker AP, Ellis IO, Elston CW, Nicholson RI and Robertson JFR (1991) An evaluation of differences in prognosis, recurrence patterns and receptor status between invasive lobular and other invasive carcinomas of the breast. *Eur J Surg Oncol* **17**: 251–257

Veldman T, Vignon C, Schrock E, Rowley JD and Ried T (1997). Hidden chromosome abnormalities in haematological malignancies detected by multicolor spectral karyotyping. *Nature Genet* **15**: 406–410

Wells WA, Rainer RO and Memoli VA (1992) Basic principles of image processing. *Am J Clin Pathol* **98**: 493–501

Yu GH, Sneige N, Kidd LD, Johnston and Katz RL (1995) Image analysis derived morphometric differences in fine needle aspirates of ductal and lobular breast carcinoma. *Anal Quant Cytol Histol* **17**: 88–92

*© Cancer Research Campaign 1999*
